# Greetings from the Editors

**DOI:** 10.1186/2191-1991-1-9

**Published:** 2011-07-26

**Authors:** J-Matthias Graf von der Schulenburg, Sebastian Braun

**Affiliations:** 1 Leibniz University Hannover, Hannover, Germany; 2 Herescon GmbH, Hannover, Germany

## 

We would like to welcome you from the editor's office of the new journal *Health Economics Review *(HER)-the first health economic e-journal. We do believe that the time is right for this type of journal in the fast growing field of health economic research. It will be a very valuable additional option for authors and readers who have an interest in health policy, public health and health economics. The character of an e-journal is that all published research articles have open-access, which means that they are immediately and permanently available online without user charge. In contrast to traditional journals-like the *European Journal of health Economics *(EJHE)-HRE is financed by fees, covered by the authors.

HRE is open for a broad range of theoretical contributions, empirical studies and analyses of health policy with a health economic focus. Its scope includes macro- and micro economics of health care financing, health insurance and reimbursement as well as health economic evaluation, health services research, pharmacoeconomics and health policy analysis. Further research topics are health care management and the growing importance of health care in developing countries.

The journal applies a web based double-blind review process. Papers will be refereed at least by two experts. However, the editors take the liberty to reject papers right away if they do not fit to the journal, arouse limited interest to the readers or do not meet scientific standards. Submissions are free of charge. The author fee will be charged only if the paper is accepted for publication.

We are very happy that we were able to engage a high ranking and active international editorial board of leading health economics experts:

Fernando Antoñanzas Villar, *University of La Rioja, Spain*

Xavier Badia, *IMS Health, Spain*

Tor Viking Eriksson, *Aarhus University, Denmark*

Steffen Fleßa, *University of Greifswald, Germany*

Ted Frech, *University of California, USA*

Wolfgang Greiner, *University of Bielefeld, Germany*

William Hsiao, *Harvard University, USA*

Panos Kanavos, *London School of Economics, UK*

Mathias Kifmann, *University of Augsburg, Germany*

Gordon G Liu, *Peking University and University of North Carolina, China*

Guillem López-Casasnovas, *University Pompeu Fabra Barcelona, Spain*

Mark J Sculpher, *University of York, UK*

Michael Stolpe, *Kiel Institute for the World Economy, Germany*

Marc Suhrcke, *University of East Anglia, UK*

Wynand PMM van de Ven, *Erasmus University Rotterdam, The Netherlands*

John Yfantopoulos, *National and Kapodistrian University of Athens, Greece*

Winnie Yip, *University of Oxford, UK*

Peter Zweifel, *University of Zurich, Switzerland*

From its start, HER-articles will be listed in the most relevant databases. In addition, we will try to receive a listing in the *Social Sciences Citation Index*^® ^as soon as possible. The sister journal, the EJHE, published by Springer as well, has currently a Social Science Citation Index of 1.34, which is expected to increase over the next years.

The editors and the editorial board guarantee, that HER articles are high-quality and peer reviewed publications. We would like to thank our colleagues in the editorial board, the first authors and referees who have contributed to a good start of the new journal and the publishing house Springer for its support. We invite everyone to submit further high-quality research manuscripts to make HER to one of the leading international journals in health economics.

J.-Matthias Graf von der Schulenburg

Editor in Chief

Sebastian Braun

Managing Editor

